# Greek Goat Encephalitis Virus Strain Isolated from *Ixodes ricinus,* Greece

**DOI:** 10.3201/eid1402.070889

**Published:** 2008-02

**Authors:** Anna Papa, Vasiliki Pavlidou, Antonis Antoniadis

**Affiliations:** * Aristotle University of Thessaloniki, Thessaloniki, Greece

**Keywords:** Greek goat encephalitis virus, tick-borne, Ixodes ricinus, ticks, dispatch

## Abstract

A strain of Greek goat encephaltitis virus was isolated from engorged *Ixodes ricinus* ticks that had fed on goats in northern Greece. The strain was almost identical to the prototype strain isolated 35 years ago.

Tick-borne encephalitis (TBE) is a zoonotic infection of the central nervous system; it is transmitted by ticks from the family Ixodidae. In an ecologic sense the disease agent, TBE virus, is an arbovirus (arthropod-borne virus); taxonomically, it is a member of the genus *Flavivirus*, family *Flaviviridae.* According to the latest taxonomy on flaviviruses, TBE virus is a species in the mammalian tick-borne virus group and has 3 subtypes: European, Far Eastern, and Siberian (*1*). Louping ill virus belongs in the same mammalian group and has 4 subtypes: British, Irish, Spanish, and Turkish. It has recently been suggested that TBE and louping ill viruses belong in the same species (TBE virus), which has 4 types: western TBE virus, eastern TBE virus (which includes Far Eastern and Siberian subtypes), Turkish sheep encephalitis virus (which includes the Greek goat encephalitis [GGE] virus,) and louping ill virus (which includes Spanish, British, and Irish subtypes) (*2*).

Information about TBE and its epidemiology in Greece is limited. The first evidence of human infection with TBE virus was reported during an investigation of the etiology of the 1927–1928 dengue epidemic. In this investigation, antibodies to TBE virus were detected by hemagglutination and neutralization tests in 1 (1.8%) of 56 serum samples (*3*). Similar results (1.7%) were found in a survey of 1,128 serum samples (*4*). In March 1969, Vergina strain (the prototype strain of GGE virus) was isolated in Vergina village, northern Greece, from the brain of a newborn goat with encephalitis-like symptoms (*5*). It was suggested at that time that GGE virus might represent a third subtype because it differed antigenically from all strains belonging to the types I and II of TBE viruses that were known at that time and transmitted by *Ixodes persulcatus* and *I. ricinus*, respectively (*6*). Because *I. gibbosus* was the only *Ixodes* spp. tick found in the region of Vergina, it has been hypothesized that GGE virus is transmitted by this species.

A serologic study, which used the hemagglutination inhibition test, of animals living permanently in northern Greece showed that 16.8% of goats, 5.6% of pigs, 5.1% of sheep, 4.7% of horses, and 3.1% of cattle had antibodies to TBE virus (*7*). A seroepidemiologic survey conducted in various prefectures in Greece found that 1.7% of the population had antibodies to TBE virus (*8*). During a recent seroepidemiologic study conducted during 2003–2005 in northern Greece, highest prevalence (5.82%) of antibodies against TBE virus was observed in the Chalkidiki prefecture (*9*). In addition, a TBE case was serologically diagnosed in this area (*10*). We report isolation of a GGE virus strain from *I. ricinus* ticks collected in Vavdos village in the Chalkidiki prefecture.

## The Study

From April through June and September through December, 2003–2006, a total of 703 adult Ixodidae ticks were collected from flocks of goats grazing permanently in 3 mountainous areas of Chalkidiki. Ticks were classified according to identification keys (Table) and grouped and assigned to pools of 10–15. Ticks were washed with sterile phosphate-buffered saline and homogenized in 500 μL of culture medium containing 4% fetal bovine serum and 500 IU/mL penicillin and streptomycin by the use of glass beads in a cell disrupter. The homogenized suspension was centrifuged at 2,500*g* for 5 min; 250 μL of the supernatant was used for RNA extraction by using TRIZOL LS Reagent (Invitrogen Life Technologies, Carlsbad, CA, USA), and the rest was stored at –70°C until further use. PCRs were performed by using 2 different pairs of primers: 1 pair of degenerated primers for the 5′ end of the envelope (E) gene (*11*) and 1 pair from the C-terminal part of the nonstructural protein 5 (NS5) gene (*12*).

**Table:** Species of ticks collected during 2004–2006, Chalkidiki, Greece

Tick species	Year			Total
	2004	2005	2006	
*Ixodes ricinus*	127	158	70	355
*Rhipicephalus bursa*	39	40	22	101
*Rh. turanicus*	25	0	0	25
*Rh. sanguineus*	76	0	0	76
*Hyalomma marginatum*	75	30	28	133
*Boophilus annulatus*	0	13	0	13
Total	342	241	120	703

One pool of *I. ricinus* ticks was TBE-positive in both reverse transcription–PCR (RT-PCR) assays. The pool consisted of 1 female and 9 male ticks collected in November 2004 in Vavdos village. Ticks of all other species were negative. Assuming that 1 tick per pool was positive, the total frequency of infected *I. ricinus* ticks was 0.28%; annual tick infection rate was 0.78% for 2004.

Of the stored supernatant, 70 μL was inoculated onto Vero E6 cells; flasks were incubated at 37°C and passaged to fresh cells every 5 days. Viral supernatants were tested by immunofluorescent assay and RT-PCR. After the fifth consecutive passage, fluorescence was present and 3 more passages of the virus (Vavdos strain) were performed.

Nucleotide sequences of the viral genes encoding 1 NS protein (NS5) as well as the capsid (C), membrane (M), and E proteins were determined by using the above-mentioned and newly designed primers (GenBank accession nos. EF693938 and EF693939). A high degree of homology with the Vergina strain (DQ235153) was observed, although the 2 strains were isolated 35 years apart and the isolation sites were 140 km apart from each other. The 2 strains differed by 2 nucleotides (0.13%) in the E gene; 1 resulted in an amino acid change (aa 122, glutamic acid in Vavdos, glycine in Vergina), but they were identical in the C, M, and NS5 genes. Similar stable phylogenetic relationships were observed in TBE virus strains of other subtypes, which indicates that the virus is remarkably stable and not subject to major antigenic variation (*13*,*14*). A probable explanation might be that TBE virus evolves within a 2-host system; furthermore, tick-borne flaviviruses evolve at 0.56 times the rate of mosquito-borne flaviviruses because of the ticks’ long life cycle and limited seasonal feeding activity (*15*).

**Figure 1:**
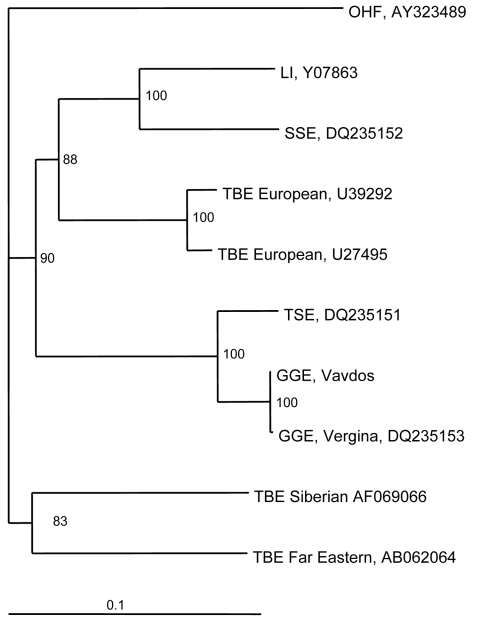
Phylogenetic tree of the envelope gene of tick-borne encephalitis (TBE) virus strains constructed by using PHYLIP software (http://evolution.genetics.washington.edu/phylip.html). Omsk hemorrhagic fever (OHF) virus was used as the out-group. The numbers at the nodes represent bootstrap values. LI, louping ill; SSE, Spanish sheep encephalitis; TSE, Turkish sheep encephalitis; GGE, Greek goat encephalitis. Scale bar, 10% nucleotide sequence divergence.

In the phylogenetic tree based on the whole E gene (1,488 nt) of TBE viruses, Greek strains cluster together with Turkish sheep encephalitis virus (nt homology 95.5%) and form an independent clade with high bootstrap value, which might represent the southern subtype (Figure 1). Genetic distances of GGE strains and those of western, eastern, and louping ill subtypes are 16.3%, 20%, and 18.5%, respectively.

**Figure 2:**
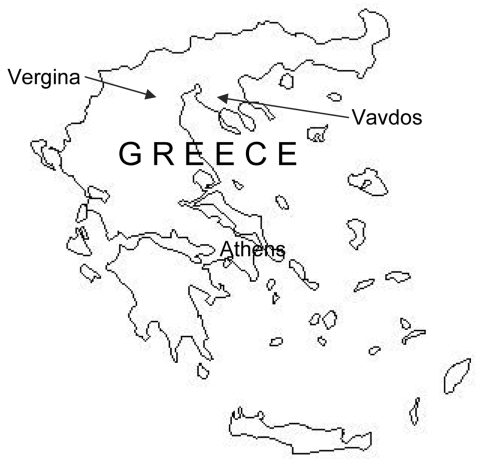
Map of Greece showing location of Vergina and Vavdos villages.

Vavdos is a mountainous village 800 m above sea level (23°26′31.1′′Ε, 40°22′8.0′′N). The area where goats were grazing was covered by typical Mediterranean low vegetation and was located at the edge of an oak forest (Figure 2). No signs of disease were present in any of the goats of the flock. In addition, the owner of the flock and his family did not report any TBE-like symptoms (but they refused to be tested for antibodies to TBE virus). The newborn goat from which the prototype GGE virus strain was isolated in 1969 had neurologic symptoms; in addition, many abortions had occurred in that flock. However, no virus was isolated from any other animal or ticks collected in northern Greece during that period, which suggests that TBE virus is rare.

## Conclusions

Natural foci of GGE virus are present in northern Greece. The strain that circulates in Greece resembles that isolated from sheep in Turkey, which has not yet been associated with disease in humans. Sequencing of the complete genome, including the more variable 3′-noncoding region, and neutralization tests that are in progress will give further insights into this group of viruses.
